# The Protective Effect of IL-17A in Pneumonic Plague Can Be Compensated by Effective Vaccines and Immunization Strategies in Mice

**DOI:** 10.3390/vaccines12121361

**Published:** 2024-12-01

**Authors:** Emily K. Hendrix, Jian Sha, Paul B. Kilgore, Blake H. Neil, Atul K. Verma, Ashok K. Chopra

**Affiliations:** 1 Department of Microbiology & Immunology, University of Texas Medical Branch, Galveston, TX 77555, USA; ekhendri@utmb.edu (E.K.H.);; 2 Institute for Human Infections and Immunity, University of Texas Medical Branch, Galveston, TX 77555, USA; 3 Sealy Institute for Vaccine Sciences, University of Texas Medical Branch, Galveston, TX 77555, USA; 4 Galveston National Laboratory, University of Texas Medical Branch, Galveston, TX 77555, USA; 5 Center for Biodefense and Emerging Infectious Diseases, Galveston, TX 77555, USA

**Keywords:** *Y. pestis*, live attenuated and adenovirus-based plague vaccines, homologous and heterologous vaccinations, IL-17A-depleted mice, flow cytometry, immunogenicity, pneumonic plague

## Abstract

Plague, caused by *Yersinia pestis*, poses a public health threat not only due to sporadic outbreaks across the globe but also due to its potential as a biothreat agent. Ironically, among the seven deadliest pandemics in global history, three were caused by *Y. pestis*. Pneumonic plague, the more contagious and severe form of the disease, is difficult to contain, requiring either prophylactic antibiotic treatment or vaccination. However, no vaccine (live attenuated or subunit) is currently approved by the Food and Drug Administration, requiring rigorous preclinical studies in different animal models, thus forming the basis of this study. **Objectives**: The aim of this study was to evaluate the efficacy and immune responses of two live attenuated vaccines (LAVs), LMA and LMP, either alone or in combination with a trivalent adenoviral vector-based vaccine (Ad5-YFV), in IL-17A-depleted and IgG control mice by using an anti-IL-17A monoclonal antibody (mAb) or its matched isotype IgG, respectively. **Methods**: IL-17A mAb or IgG isotype control was administered to mice twice per week to their respective groups during the course of immunization. Serum, spleens, and broncho-alveolar lavage fluid (BALF) were collected for assessing immunological responses, and another cohort of mice was intranasally challenged with a lethal dose of parental *Y. pestis* CO92. **Results**: Robust humoral and cellular immune responses followed by complete protection were observed in all vaccinated animals against highly lethal intranasal challenge doses of parental *Y. pestis* CO92. Serum IgG titers to YscF and overall mucosal IgA titers to all three antigens of the Ad5-YFV vaccine were significantly lower, with slightly reduced serum LcrV-neutralizing antibodies when IL-17A was depleted compared to IgG control animals during the course of immunization. A remarkable reduction in Th1 (IFNγ or IL-2) and Th17 cell populations was observed in IL-17A-depleted mice compared to IgG controls in response to vaccination. On the other hand, B cell activities in germinal centers, overall activated antigen-specific T cells, and memory B and T cells remained at comparable levels in both vaccinated IL-17A-depleted and IgG control mice. **Conclusions**: These data demonstrated the effectiveness of our vaccines even under the reduced levels of both Th1 and Th17 responses and thus should be suitable for those individuals associated with certain immune deficiencies.

## 1. Introduction

Plague, the disease caused by *Yersinia pestis*, has resulted in three pandemics with a staggering number of deaths exceeding 200 million [1]. Plague continues to re-emerge, especially in regions of endemicity. A 2017–2018 plague epidemic in Madagascar resulted in more than 2500 cases and over 200 deaths [2]. Significantly, the majority of cases in this epidemic were pneumonic rather than the more common bubonic manifestation [2]. Pneumonic plague is typically much more severe and has a much higher fatality rate than bubonic plague, approaching 100% if left untreated [2]. In addition, antibiotic-resistant strains of *Y. pestis* have emerged [3], making treatment strategies difficult. Therefore, vaccination remains the most effective approach in preventing plague transmission. There are, however, no Food and Drug Administration (FDA)-approved plague vaccines [4]. 

Earlier plague vaccines were either heat- or formaldehyde-inactivated *Y. pestis* bacilli. The use of these vaccines was discontinued largely due to severe side effects and limited efficacy against pneumonic plague [2]. Subsequently, live attenuated [5,6,7,8], vector-based (bacterial or viral) [9,10,11,12,13], recombinant [14,15], DNA [16], or the most recent messenger RNA (mRNA)-based [17,18] strategies have been employed for plague vaccine development. The advantages and disadvantages of these vaccines have been detailed by us and other researchers in several review articles [2,19,20].

A vast majority of subunit and vector-based plague vaccines in preclinical and clinical development are based on a combination of the fraction 1 capsule-like antigen (F1) and the low-calcium-response V antigen (LcrV) on the needle tip of the type 3 secretion system (T3SS) [2,21,22,23,24,25,26,27,28,29]. However, *Y. pestis* strains that lack the F1 capsule (F1^-^) and remain virulent have been reported, and LcrV alone does not provide adequate protection against infection [9,30,31]. We have generated multiple vaccine candidates, two live attenuated vaccines (LAVs) and one adenovirus-based vaccine, that have been characterized in detail, overcoming some drawbacks encountered with other vaccine candidates. The LAVs are triple-deletion mutants of *Y. pestis* strain CO92 deleted for genes encoding Braun lipoprotein (Lpp), acetyltransferase B (MsbB), and attachment-invasion locus (Ail), designated as LMA, or deletion of Lpp, MsbB, and plasminogen-activating protease (Pla), designated as LMP. Both LMA and LMP were avirulent while remaining highly immunogenic in mouse and rat models [7,8,32,33,34] and are classified as exempt strains by the Centers for Disease Control and Prevention (CDC) (https://www.selectagents.gov/sat/exclusions/hhs.htm; accessed 29 November 2023) and the National Institutes of Health (NIH). Hence, these can now be used at the lower biosafety level BSL-2/ABSL-2. The adenovirus-based vaccine, Ad5-YFV, is based on a replication-defective human adenovirus type 5 (Ad5) encompassing a fusion gene cassette for three prominent plague antigens: the *Yersinia* secretion protein F (YscF) of the T3SS barrel, F1, and LcrV [10,35,36]. Incorporating YscF into this vaccine platform compensates for the lack of protection seen with the Ad5-LcrV monovalent vaccine or other F1V vaccine candidates against *Y. pestis* strains lacking the F1 capsule [9]. 

The implication of the IL-17 pathway in an acute pneumonic plague mouse model was first shown in 2007, when elevated production of IL-17A and its promoting cytokines, such as transforming growth factors beta (TGF-β), interleukin-6 (IL-6), tumor necrosis factor alpha (TNF-α), and IL-1β, were observed in inflamed lungs [37]. Subsequently, IL-17A produced from innate immune cells, especially neutrophils, was shown to have a protective role against pneumonic infection in mice through orchestrating interferon gamma (IFNγ)-activated macrophage programming [38]. Furthermore, vaccinating B cell-deficient mice with D27-pLpxL, a live attenuated *Y. pestis* strain, induced cell-mediated protection against lethal pulmonary *Y. pestis* infection, and prime-boost immunization conferred better protection than prime-only vaccination of mice [39]. Importantly, this improved survival did not result from enhanced bacterial clearance but was associated with increased levels of IL-17A mRNA and protein in the lungs of challenged mice. These data suggested that an optimal plague vaccine should also be able to elicit a Th17 response in addition to a Th1 response [39]. Consistent with this, vaccinating mice and rats with our two LAVs, either alone or in combination with Ad5-YFV, also induced significantly higher levels of both Th1 and Th17 responses during our previous studies [8,40]. To further assess the role of IL-17A on the efficiency of our vaccines, we evaluated both humoral and cellular immunity in IL-17A-depleted mice. Complete protection was observed in all immunized mice against lethal pulmonary *Y. pestis* challenge, irrespective of whether IL-17A was depleted or not. Of note, IL-17A influenced the production of both humoral and cellular immunity. To our knowledge, this is the first detailed immunogenicity and challenge study of plague vaccines in IL-17A-depleted mice. 

## 2. Materials and Methods

### 2.1. Animals

Six-week-old female Swiss Webster mice were purchased from Jackson Laboratory (Bar Harbor, ME). All studies were ethically performed under an approved Institutional Animal Care and Use Committee (IACUC) protocol (Office of Laboratory Animal Welfare assurance number A3314-01).

### 2.2. In Vivo Treatment with Antibodies

For IL-17A depletion, mice were injected intraperitoneally (i.p.) in the lower right quadrant with anti-IL-17A monoclonal antibody (mAb; 150 µg/mouse/treatment; BioXCell, Lebanon, NH, USA; Clone 17F3) twice per week over the duration of vaccination before harvesting splenocytes and challenge. The corresponding isotype control antibodies were similarly used (Mouse IgG1; BioXCell; Clone MOPC-21). The concentrations of mAbs used were based on the manufacturer’s instructions.

### 2.3. Bacterial Strains and Vaccines

The human *Y. pestis* isolate CO92 was obtained from BEI Resources (Manassas, VA, USA). The live attenuated vaccine candidates LMA and LMP are triple-deletion mutants (Δ*lpp*Δ*msb*BΔ*ail* [LMA] and Δ*lpp*Δ*msbB*Δ*pla* [LMP]) of CO92, deleted for genes encoding Braun lipoprotein (Lpp), acetyltransferase B (MsbB), and attachment-invasion locus (Ail) or plasminogen-activating protease (Pla), respectively [8,32]. As we previously described [40], LMA and LMP were prepared by inoculating heart infusion broth (HIB) in an overnight shaking culture (28 °C, 180 rpm). The LAVs were then aliquoted into 500 µL volumes at 1 × 10^9^ CFU/mL with 25% glycerol and stored at −80 °C. For vaccination, the stored LAVs were further diluted to the desired concentration in PBS. The replication-deficient human adenoviral type 5 vector-based vaccine Ad5-YFV includes genes for three plague antigens: YscF (TS barrel), F1 (capsule), and LcrV (T3SS needle) [10]. The Ad5-YFV vaccine was purified at the Baylor College of Medicine Vector Development Laboratory and at Lonza (Houston, TX) under GLP conditions. Ad5-YFV was aliquoted at 1 × 10^12^ v.p./mL and stored at −80 °C. Titration was routinely confirmed before and after each inoculation. All studies utilizing *Y. pestis* were performed in Tier 1 select agent BSL-3 laboratories at UTMB in the GNL-Keiller complex in Galveston, Texas. All animal studies were conducted in ABSL-3 facilities of the GNL-Keiller complex.

### 2.4. Vaccination and Challenge Studies

During IL-17A depletion, mice were anesthetized by 2–4% isoflurane inhalation and a 2 L/min O_2_ flow rate in an induction chamber (VetEquip, Pleasanton, CA, USA) and immunized with two doses of LMA or LMP alone intramuscularly (i.m., 2 × 10^6^ CFU/50 µL) in the hind limb, or in combination with Ad5-YFV intranasally (i.n., 5.5 × 10^10^ PFU/50 µL). Vaccines were administered 21 days apart. PBS was administered to animals as unvaccinated controls.

Mice (*n =* 10 per group per IL-17A or IgG mAb treatment) were anesthetized by isoflurane inhalation and retro-orbitally bled. Serum samples were pooled prior to vaccination or challenge and around 20 days after each immunization. On day 39, a cohort with 5 mice from each group was anesthetized by isoflurane inhalation and challenged i.n. with 100 LD_50_ of *Y. pestis* CO92. Seven days post-initial challenge, surviving mice and naïve age-matched control mice were re-challenged i.n. with 10,000 LD_50_ of *Y. pestis* CO92. On day 49, animals were humanely euthanized using i.p. injection of 5–10 mg/kg ketamine and xylazine in 100 µL followed by cervical dislocation. Spleens and broncho-alveolar lavage fluid (BALF) were collected individually from a separate cohort with the remaining 5 mice in each group for immunological analysis.

### 2.5. Antibody Titers 

Antibody titers were determined via ELISA as we previously described [40]. Briefly, MaxiSorp Nunc ELISA plates (Fisher Scientific, Newington, NH, USA) were coated with 100 ng/100 µL of recombinant fusion protein (rF1V, BEI Resources, Manassas, VA, USA) or individual antigens (rF1, rLcrV, or rYscF) in carbonate buffer and incubated overnight at 4°C. Plates were washed with Dulbecco’s PBS (DPBS) containing 0.05% Tween 20 and then blocked with 1% bovine serum albumin (BSA) (Sigma Aldrich, St. Louis, MO, USA). Serum and BALF were 2-fold serially diluted and added to plates for 1 h at room temperature. Plates were then washed and horseradish peroxidase (HRP)-conjugated secondary anti-mouse antibodies for IgG, IgG1, IgG2c, or IgA (Southern Biotech, Birmingham, AL, USA) diluted to 1:8000 were added. Plates were washed and 3,3’,5,5’-tetramethyl-benzidine (TMB) substrate was added. The reaction was stopped using 2N H_2_SO_4_, and absorbance was measured at 450 nm using a VersaMax tunable microplate reader (Molecular Devices, San Jose, CA, USA). Total IgG, IgG1, IgG2c, and IgA were measured in triplicate. 

### 2.6. Neutralization Antibody Analysis

LcrV Mab7.3 was biotinylated using EZ-Link™ Sulfo-NHS Biotin (ThermoFisher, Waltham, MA, USA) [41] and purified with Sephadex G-25 resin PD-10 desalting columns (Cytiva, Marlborough, MA, USA) following the manufacturer’s instructions. The biotinylated antibody was eluted in PBS, and its concentration was determined using a Nanodrop™ One Spectrophotometer (Invitrogen, Carlsbad, CA, USA) at A280. Neutralization antibodies were measured using competitive ELISA [42] and completed as follows: Maxisorp Nunc ELISA plates were coated with 200 ng of recombinant LcrV (rV) (BEI) in 100 μL of PBS overnight at 4 °C. Unbound antigens were removed by three washes with Dulbecco’s PBS (DPBS) containing 0.05% Tween 20. Plates were blocked with 150 µl of 2% skim milk powder (Millipore Sigma, Burlington, MA, USA) in PBS for 1 h at room temperature. After washing, wells were incubated for 2 h with 80 ng/well of biotinylated LcrV Mab7.3 in 100 μL of blocking buffer. After washing, heterologously vaccinated IL-17A-depleted mouse serum was diluted 1:10 and then 2-fold serially diluted and incubated for 2 h at room temperature. After washing, 100 μL of 1:10,000 streptavidin-HRP conjugate was added for 45 min and developed in 100 μL of TMB. Development of the colorimetric reaction was stopped using 2N H_2_SO_4_ and absorbance was measured at 450 nm using a VersaMax tunable microplate reader. The dilution of the test serum that inhibited binding of the biotinylated Mab7.3 to rV by 50% or greater (NT_50_) represented the level of neutralizing antibodies.

### 2.7. Flow Cytometry Analysis

T cell phenotypes: Spleens were collected and processed as previously described [40]. After 2 days of stimulation with 100 µg/mL of rF1V antigen (BEI Resources), 1X Brefeldin A was added for an additional 4 h. Splenocytes were then harvested, blocked with anti-mouse CD16/32 antibodies (Biolegend, San Diego, CA, USA), and stained with fixable viability dye eFluor 780 (Invitrogen), Alexa Fluor 700 anti-mouse CD3 (Biolegend), Brilliant Violet 785 anti-mouse CD4 (Biolegend), FITC anti-mouse CD8 (Biolegend), Brilliant Violet 510 anti-mouse CD44 (Biolegend), Brilliant Violet 711 anti-mouse CD62-L (Biolegend), PE anti-mouse CD127 (Biolegend), Brilliant Violet 605 anti-mouse CD25 (Biolegend), and APC anti-mouse CD134 (Biolegend). Cells were permeabilized for intracellular staining with the Foxp3/Transcription Factor Staining Buffer Set (eBioscience, San Diego, CA, USA) at 4 °C overnight and then stained with Brilliant Ultra Violet anti-mouse CD69 (BD Biosciences, San Jose, CA, USA), PE/Cyanine7 anti-mouse IL-17A (Biolegend), PerCP/Cyanine5.5 anti-mouse IFNγ (Biolegend), eFluor 450 anti-mouse TNFα (eBioscience), PE-CF594 anti-mouse IL-2 (BD Biosciences), and Brilliant Violet 650 anti-mouse IL-4 (BD Biosciences) and analyzed via flow cytometry as described in our previous studies [7,9,40] on the BD FACSymphony A5.

B cell phenotypes: Splenocytes isolated from the above mice were directly subjected to B cell panel staining without rF1V stimulation. Briefly, splenocytes (2 × 10^6^ cells) were blocked with anti-mouse CD16/32 (Biolegend) antibodies followed by staining with fixable viability dye eFluor 780 (Invitrogen), FITC anti-mouse CD19 (Biolegend), Alexa Fluor 700 anti-mouse CD3 (Biolegend), Brilliant Violet 650 anti-mouse CD138 (BD Biosciences), PE-Cy7 anti-mouse CD38 (Biolegend), PerCP/Cyanine 5.5 anti-mouse GL7 (Biolegend), Brilliant Violet 510 anti-mouse IgD (Biolegend), Brilliant Violet 421 anti-mouse CD80 (Biolegend), Brilliant Violet 605 anti-mouse CD73 (Biolegend), and PE anti-mouse PDL2 (Biolegend), and analyzed via flow cytometry on the BD FACSymphony A5. Flow cytometry data were prepared via FlowJo software v10.10.

### 2.8. Statistical Analysis

All outcomes were statistically compared using appropriate parametric and non-parametric tests such as Kaplan–Meier with Mantel–Cox tests for survival. *In vitro* experiments were performed in triplicate. Statistical analysis between groups was conducted using one- and two-way analysis of variance (ANOVAs). Results were plotted in GraphPad PRISM 9 software, with results expressed as means ± standard deviation (SD). *p*-values < 0.05 were considered significant.

## 3. Results

### 3.1. Vaccinated Animals Are Protected Against Pneumonic Plague

**Figure 1:**
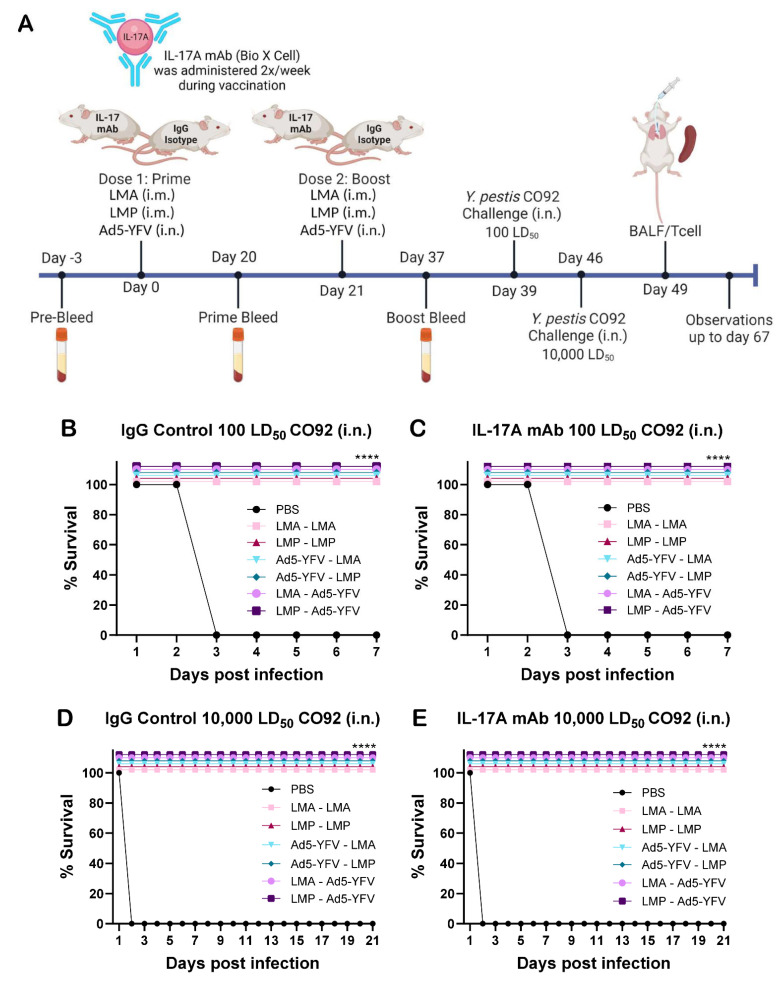
Vaccination protected both IL-17A-depleted and IgG control mice against lethal pulmonary *Y. pestis* challenge. (**A**) Experimental time course. Swiss Webster mice (*n =* 10 per group) administered anti-IL-17A or its IgG isotype control mAbs twice per week were immunized with either LMA or LMP alone by i.m. injection or in combination with i.n. instillation of Ad5-YFV in a 2-dose regimen, 21 days apart. Mice receiving PBS were used as challenge controls. (**B**,**C**) Approximately 3 weeks after the completion of the vaccination schedule, mice were i.n. challenged with 100 LD_50_ of *Y. pestis* CO92. (**D**,**E**) Seven days post-initial challenge, survivors among vaccinated mice, along with age-matched naïve control mice, were re-challenged i.n. with 10,000 LD_50_ of *Y. pestis* CO92 and observed for morbidity and mortality for 21 days. Kaplan–Meier analysis with log-rank (Mantel–Cox) test was used to analyze survival. Asterisks represent the statistical significance of vaccinated groups compared to naïve control mice. **** *p* < 0.0001.

To deplete IL-17A, anti-IL-17A monoclonal antibodies (mAbs) were administered to mice twice per week by the intraperitoneal (i.p.) route during the entire course of immunization, and isotype control IgG was injected into control animals. Both IL-17A-depleted and IgG control mice were vaccinated either intramuscularly (i.m.) with LMA or LMP individually (homologous strategy) or in combination with intranasal (i.n.) delivery of Ad5-YFV (heterologous strategy). Vaccines were administered in a prime-boost regimen 21 days apart, with prime-pull vaccinations being those where Ad5-YFV was administered as the second dose in a heterologous strategy (Figure 1A). During the depletion and immunization course, we did not observe any local or systemic adverse effects, such as weight loss, ruffled fur, or injection site redness in either IL-17A-depleted or IgG control mice. Roughly 3 weeks post-boost vaccination, mice were i.n. challenged first with 100 LD_50_ of *Y. pestis* CO92, where 1 LD_50_ is 500 colony-forming units (CFU) of CO92 in Swiss Webster mice [30,43]. All vaccination strategies, regardless of the order or combination, provided 100% protection to immunized mice, irrespective of the status of IL-17A depletion, while 100% lethality was observed in unvaccinated control mice (given phosphate-buffered saline [PBS] as a vehicle) 3 days post-infection (d.p.i.) (Figure 1B,C). Seven days post-initial challenge, surviving vaccinated mice and new age-matched naïve control animals were re-challenged i.n with 10,000 LD_50_ of *Y. pestis* CO92 and observed over 21 days. All vaccination strategies provided 100% protection to mice, while 100% lethality was observed in challenge control mice 2 d.p.i. (Figure 1D,E). Secondary challenge at an extremely high dose of *Y. pestis* in vaccinated mice not only mimics the accumulation of *Y. pestis* bacilli in the environment during plague outbreaks but also showcases the efficacy of the recall immune response in immunized animals, which usually reaches its peak around 7–10 days post-challenge. Vaccinated mice exhibited no significant clinical signs, such as weight loss, ruffled fur, or conjunctivitis, post-challenge compared to unimmunized animals.

### 3.2. Vaccinated Mice Exhibit Increased Antibody and B Cell Responses

**Figure 2:**
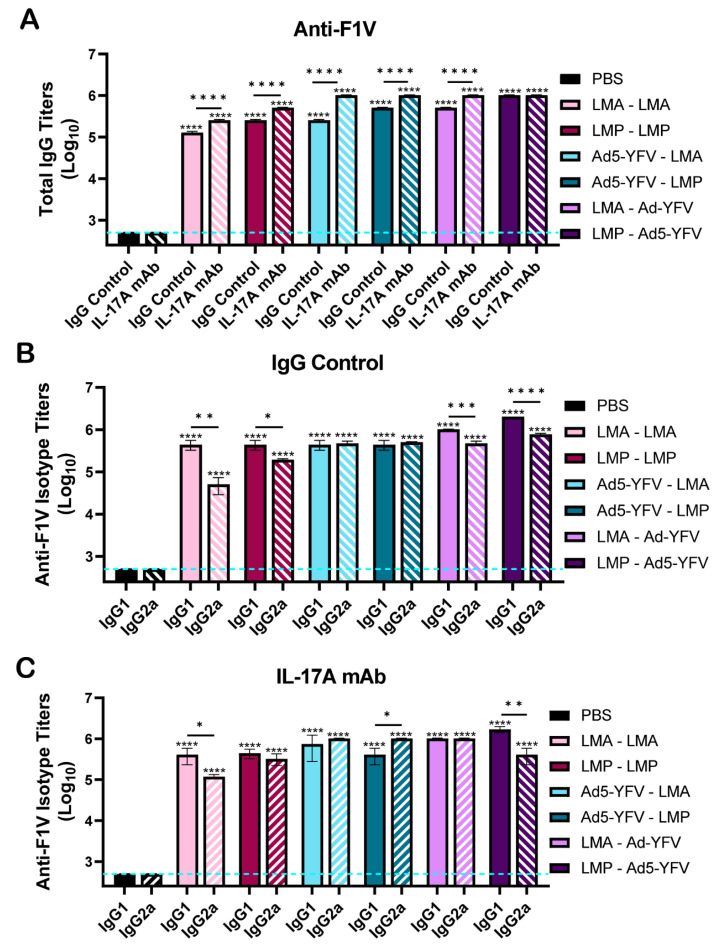
Serum IgG titers and their sub-isotypes to the F1V fusion protein in vaccinated IL-17A-depleted and IgG control mice. Boost sera were collected on day 37 as described in Figure 1A and ELISAs were performed to evaluate (**A**) total IgG and (**B**,**C**) IgG isotype titers to F1V. Significance was determined by either one-way ANOVA with Tukey’s post hoc test (**A**) or two-way ANOVA with Tukey’s post hoc test (**B**,**C**). The geometric means ± standard deviations are plotted. The blue dashed line represents the baseline values of naïve control samples at the dilution of 1:500. Asterisks with comparison bars represent statistical significance between respective vaccinated groups, while asterisks directly above the bars represent statistical significance of vaccinated groups compared to naïve control mice. *, *p* < 0.05; **, *p* < 0.01; ***, *p* < 0.001; ****, *p* < 0.0001. These data were combined from three independent ELISA assays.

Serum was collected prior to challenge with *Y. pestis* CO92, around day 20 (prime) and 16 days post-boost immunization (day 37 of the study) (Figure 1A). Both IL-17A-depleted and IgG control mice elicited significantly increased IgG titers to the recombinant F1 and LcrV fusion protein (rF1V) compared to unvaccinated control animals, irrespective of the vaccination combination (Figure 2A). F1V IgG titers increased by up to 1 log from the prime to boost vaccination. Compared to IgG control mice, significantly higher F1V IgG titers were noted in IL-17A-depleted mice, except for LMP–Ad5-YFV vaccinated animals, in which a comparable level of F1V IgG was observed. On the other hand, slightly lower F1V IgG titers were noted in homologously vaccinated mice compared to heterologously vaccinated animals in both IL-17A-depleted and IgG control mice, suggesting that the heterologous strategy of vaccination produced improved humoral responses compared to the homologous strategy (Figure 2A). We next examined IgG antibody isotypes, IgG1 and IgG2a, in the serum of vaccinated mice to evaluate the T helper 1 (Th1) versus Th2 bias. Vaccinated mice generally showed an overall balanced F1V-specific Th1 vs. Th2 response; however, animals that received LAVs as the first dose, irrespective of IL-17A depletion status, were slightly skewed to a Th2 response with higher IgG1 over IgG2a. In contrast, animals vaccinated first with Ad5-YFV had either equal levels of IgG1 and IgG2a (in IgG control mice) or slightly higher IgG2a (in IL-17A-depleted mice), representing a more balanced or Th1 response (Figure 2B,C). These data suggested that the types of Th responses developed were likely associated with the types of vaccines and the influence of IL-17A on antibody isotype class switching appeared minimal.

**Figure 3:**
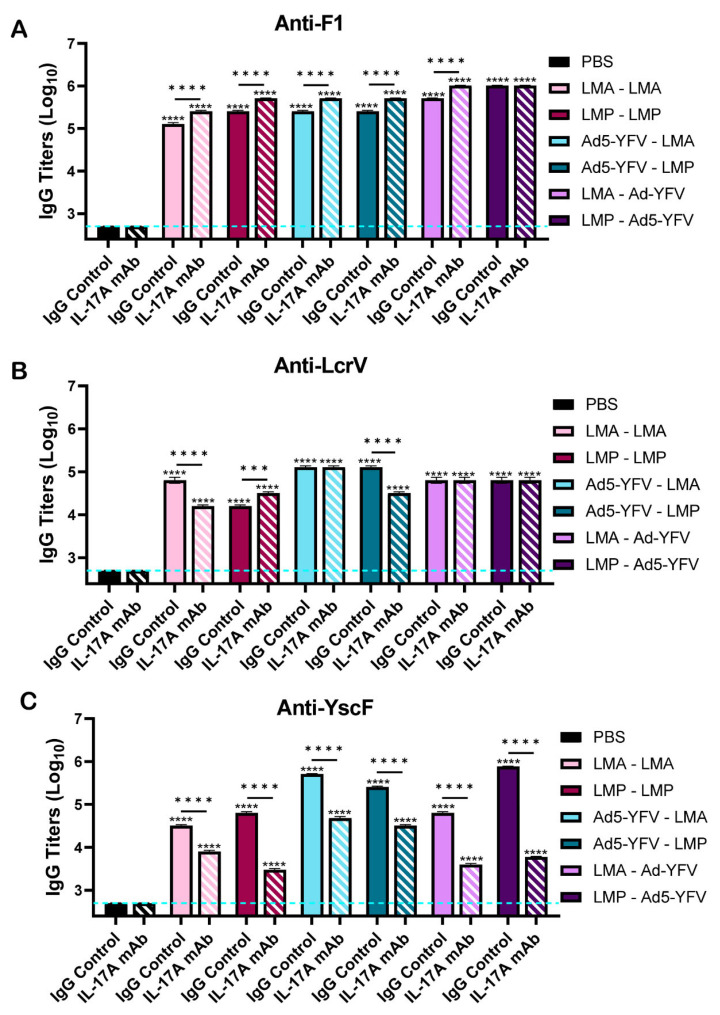
Serum IgG antibody responses to specific individual plague antigens in vaccinated IL-17A-depleted and IgG control mice. Boost sera were collected on day 37 as described in Figure 1A and ELISAs were performed to evaluate IgG titers to individual plague antigens (**A**) F1, (**B**) LcrV, and (**C**) YscF. The geometric means ± standard deviations are plotted. Statistical significance was determined by one-way ANOVA with Tukey’s post hoc test. The blue dashed line represents the baseline values of naïve control samples at the dilution of 1:500. Asterisks with comparison bars represent statistical significance between respective vaccinated groups, while asterisks directly above the bars represent statistical significance of vaccinated groups compared to naïve control mice. *** *p* < 0.001, **** *p* < 0.0001. These data are combined from three independent ELISA assays.

To further analyze the humoral response in vaccinated mice, IgG titers in boost sera (day 37) to three individual plague-specific antigens F1, LcrV, and YscF, constituents of the Ad5-YFV vaccine, were assessed. Overall, all vaccinated mice, irrespective of IL-17A depletion status, demonstrated significantly higher anti-F1, -LcrV, and -YscF serum IgG titers compared to unvaccinated control mice (Figure 3A–C). Antibody titers were highest to F1, indicating its dominance in the serum and reflecting antibody titers to the F1V fusion antigen. The F1 IgG titers were significantly higher in IL-17A-depleted mice than in IgG control animals (Figure 2A and Figure 3A). In contrast, vaccinated IL-17A-depleted mice developed significantly lower YscF-specific IgG titers compared to vaccinated IgG controls (Figure 3C). The influence of IL-17A on the production of LcrV antibodies varied and seemed dependent on the vaccines that mice received. More specifically, compared to immunized IL-17A-depleted mice, higher anti-LcrV titers were observed in IgG control mice vaccinated with either LMA-LMA or Ad5-YFV–LMP. On the other hand, a lower anti-LcrV titer was noted when IgG control mice were immunized with LMP-LMP (Figure 3B). 

**Figure 4:**
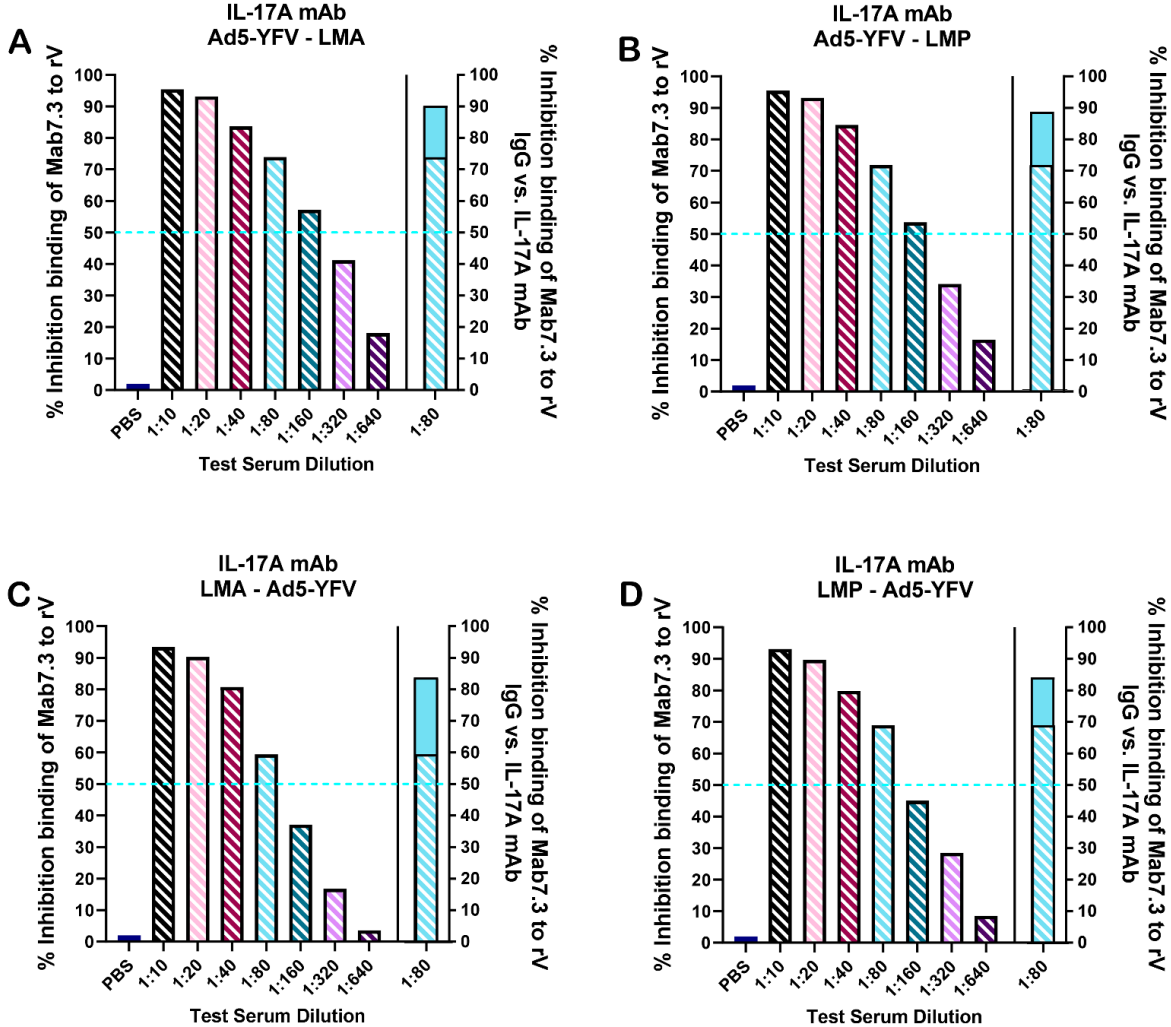
Neutralizing antibodies in vaccinated IL-17A-depleted mice. Animals were immunized and serum was collected as described in Figure 1A. Competitive ELISAs were performed using biotinylated neutralizing and protective LcrV Mab7.3 against serum from animals vaccinated with (**A**) Ad5-YFV–LMA, (**B**) Ad5-YFV–LMP, (**C**) LMA–Ad5-YFV, or (**D**) LMP–Ad5-YFV in binding of rV. The percentage inhibition of biotinylated LcrV Mab7.3 binding to rV by immunized sera was plotted. The blue dashed line represents 50% of the biotinylated LcrV Mab7.3 outcompeted for binding. The area to the left of the vertical solid line depicts the competitive binding capacity of sera at 2-fold increasing dilutions. The area to the right of the vertical solid line depicts the comparison of competitive binding ability of serum at a 1:80 dilution with biotinylated LcrV Mab7.3 between IL-17A-depleted (striped blue bar) and IgG control (solid blue bar) groups. Averages from representative experiments are plotted.

To gauge the level of neutralizing antibodies in the serum of vaccinated mice, competitive ELISAs were performed, whereby biotinylated LcrV Mab7.3, shown to passively protect mice against both bubonic and pneumonic plague [44], was used. As depicted in Figure 4A,B, heterologous vaccination strategies resulted in the generation of serum antibodies capable of competitively binding to the rV epitope specific for LcrV Mab7.3. These antibody titers were slightly higher when the Ad5-YFV vaccine was administered first. Fifty percent of biotinylated LcrV Mab7.3 was outcompeted roughly at a serum dilution of 1:240 when Ad5-YFV was administered first, compared to roughly 1:120 when LAVs were administered first (Figure 4C,D). Subsequently, we compared the ability of serum at a dilution of 1:80 to outcompete biotinylated LcrV Mab7.3 between IL-17A-depleted and IgG control mice. As noted in Figure 4, the ability of serum antibodies to competitively bind was slightly lower when IL-17A was depleted in vaccinated mice (striped blue bar), compared to vaccinated IgG control animals (solid blue bar).

**Figure 5:**
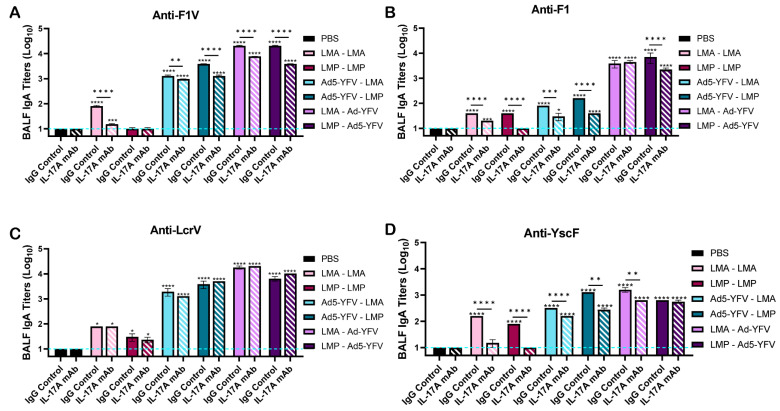
BALF IgA titers in vaccinated IL-17A-depleted and IgG control mice. Animals were immunized and BALF was collected as described in Figure 1A. IgA ELISAs were performed to evaluate titers to (**A**) F1V fusion plague antigen and individual plague antigens (**B**) F1, (**C**) LcrV, and (**D**) YscF. The geometric means ± standard deviations are plotted. Statistical significance was determined by one-way ANOVA with Tukey’s post hoc test. The blue dashed line represents the baseline values of naïve control samples at the dilution of 1:500. Asterisks with comparison bars represent statistical significance between respective vaccinated groups, while asterisks directly above the bars represent statistical significance of vaccinated groups compared to naïve control mice. * *p* < 0.05, ** *p* < 0.01, *** *p* < 0.001, **** *p* < 0.0001. These data are combined from three independent ELISA assays.

To evaluate mucosal immunity, we examined IgA antibody titers in broncho-alveolar lavage fluid (BALF). Overall, immunized mice developed higher IgA titers than unvaccinated animals (Figure 5A–D). Importantly, IgA antibody titers to all examined antigens in vaccinated IL-17A-depleted mice were either significantly lower or at similar levels compared to immunized IgG control animals (Figure 5A–D). This indicated that IL-17A influenced mucosal antibody production as opposed to serum IgG titers, where the influence of IL-17A was most likely dependent on the antigens themselves (Figure 3A–C). Importantly, irrespective of IL-17A depletion status, the highest IgA titers were observed in prime-pull immunized animals, followed by mice receiving other heterologous vaccinations. Mice receiving LAVs had the lowest IgA titers in BALF (Figure 5A–D).

**Figure 6:**
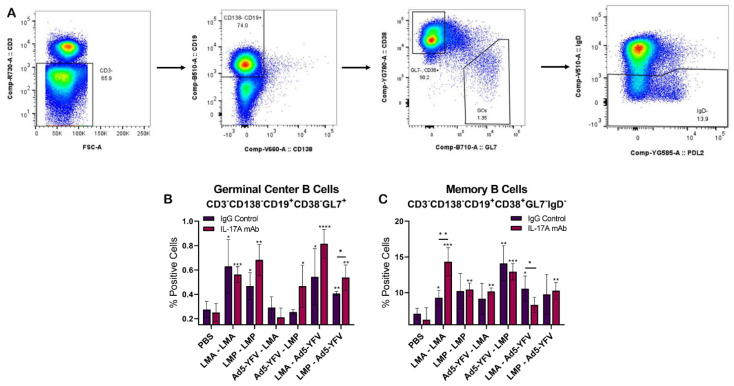
Germinal center B cell activities and memory B cell populations in vaccinated IL-17A depleted and IgG control mice. Spleens were collected 4 weeks post-boost immunization from vaccinated and naïve control mice (*n* = 5 per group). (**A**) Splenocytes were surface-stained with various B cell markers and subjected to flow cytometry. The percentage of B cells in (**B**) germinal centers was gated as CD3^-^CD138^-^CD19^+^CD38^-^GL7^+^, while (**C**) memory B cells were identified as CD3^-^CD138^-^CD19^+^CD38^+^GL7^-^IgD^-^. The arithmetic means ± standard deviations are plotted. Statistical significance was determined using one-way ANOVA with Tukey’s post hoc test. Asterisks with comparison bars represent statistical significance between respective vaccinated groups, while asterisks directly above the bars represent the statistical significance of vaccinated groups compared to naïve control mice. *, *p* < 0.05; **, *p* < 0.01; ***, *p* < 0.001; ****, *p* < 0.0001.

To further examine humoral immune responses, splenocytes were isolated from vaccinated IL-17A-depleted, IgG control, and unvaccinated control animals (Figure 1A). Cells were stained for germinal center activity (CD3^-^CD138^-^CD19^+^CD38^-^GL7^+^) and memory B cells (CD3^-^CD138^-^CD19^+^GL7^-^CD38^+^IgD^-^) (Figure 6A). Compared to unvaccinated controls, both vaccinated IL-17A-depleted and IgG control animals displayed an overall increase in germinal center B cells, with a few exceptions (Figure 6B), and memory B cells (Figure 6C), although some did not reach statistically significant levels, and their levels were generally comparable irrespective of IL-17A depletion status. The GC B cell population between IL-17A-depleted and IgG controls only reached significance when animals were vaccinated with LMP-Ad5-YFV (Figure 6B). Likewise, statistical differences in memory B cells between IL-17A-depleted and IgG controls were only observed when animals were vaccinated with either LMA-LMA or LMA–Ad5-YFV (Figure 6C). 

### 3.3. T Cell Responses Are Induced by Vaccination

**Figure 7:**
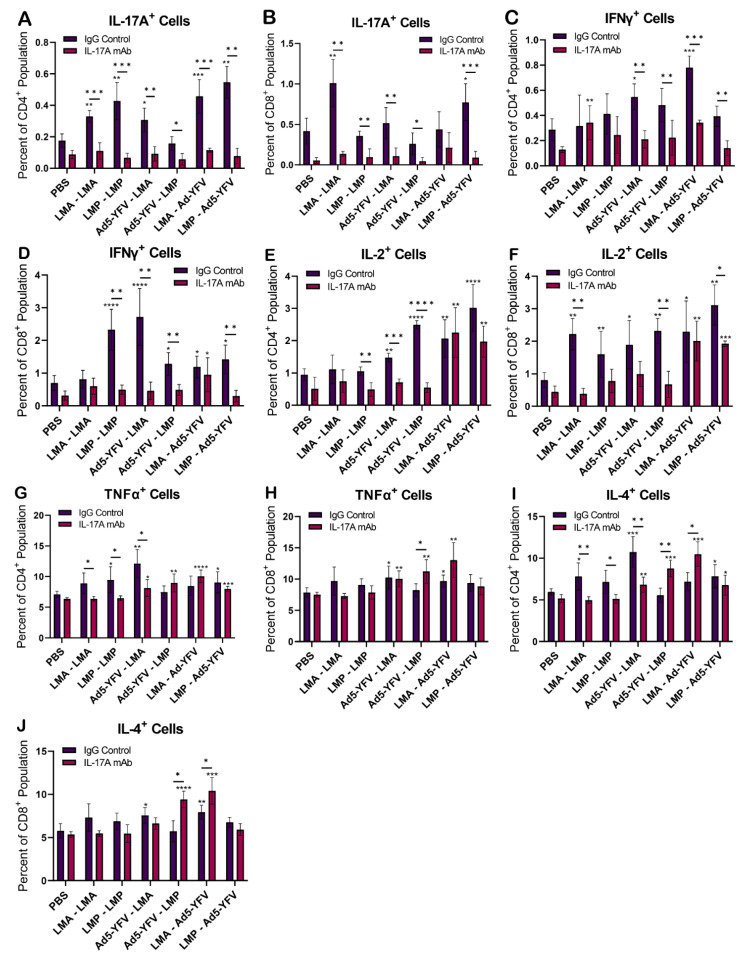
Cytokine-positive T cells in vaccinated IL-17A-depleted and IgG control mice. Spleens were collected 4 weeks post-boost immunization from vaccinated and naïve control mice (*n =* 5 per group). The isolated splenocytes were stimulated with rF1V (100 µg/mL) for 48 h at 37°C, followed by an additional 4 h with Brefeldin A. Splenocytes were surface- and intracellularly stained with various cytokine and T cell markers and subjected to flow cytometry. (**A**,**C**,**E**,**G**,**I**). The percentages of cytokine-positive CD4^+^ and (**B**,**D**,**F,H**,**J**) CD8^+^ populations were acquired. The arithmetic means ± standard deviations are plotted. Statistical significance was determined using one-way ANOVA with Tukey’s post hoc test. Asterisks with comparison bars represent statistical significance between respective vaccinated groups, while asterisks directly above the bars represent the statistical significance of vaccinated groups compared to naïve control mice. * *p* < 0.05, ** *p* < 0.01, *** *p* < 0.001, **** *p* < 0.0001.

To examine the cell-mediated response, splenocytes were isolated from IL-17A-depleted, IgG control, and unvaccinated control animals 4 weeks post-boost vaccination (Figure 1A) and stained for anti-CD3, -CD4, and -CD8 followed by intracellular staining for various cytokines (IFNγ, IL-2, TNFα, IL-4, and IL-17A). Compared to unvaccinated control animals, IL-17A^+^ T cells (both CD4^+^ and CD8^+^) were at basal levels in immunized and IL-17A-depleted mice, while they were significantly increased in all vaccinated IgG control animals. The highest IL-17A^+^ CD4^+^ population was observed in IgG control animals vaccinated by the prime-pull strategy (Figure 7A). A similar trend was also observed for IFNγ^+^ and IL-2^+^ T cells in response to vaccination, which were overall much lower in IL-17A-depleted animals compared to IgG controls (Figure 7C–F). However, this trend was not observed in TNFα^+^ T cell populations, as comparable levels were observed between some IL-17A-depleted and IgG control animals in response to vaccination. Furthermore, the overall TNFα^+^ T cell response was only slightly to moderately increased in immunized animals compared to unimmunized controls (Figure 7G–H). Interestingly, the production of Th1 cytokine-producing (IL-2 and TNFα) T cells was partially recovered in IL-17A-depleted animals receiving prime-pull vaccinations, reaching significance compared to unimmunized control mice (Figure 7E–H). 

Similarly, the overall IL-4^+^ T cell response was only slightly to moderately increased in immunized animals compared to unimmunized controls (Figure 7I,J). A significantly higher percentage of IL-4^+^ CD4^+^ cells in IgG control mice immunized with either LAVs or Ad5-YFV—LMA was observed compared to IL-17A-depleted mice, but the opposite pattern was observed in mice vaccinated with Ad5-YFV–LMP or LMA–Ad5-YFV (Figure 7I). Likewise, the IL-4^+^ CD8^+^ population was significantly increased in IL-17A-depleted mice compared to IgG control mice in response to immunization with either Ad5-YFV–LMP or LMA–Ad5-YFV (Figure 7J).

**Figure 8:**
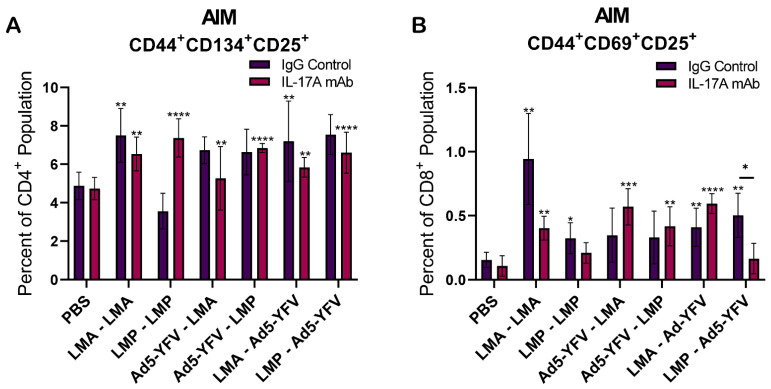
Evaluation of T cell response through the activation-induced marker (AIM) assay. Spleens were collected 4 weeks post-boost immunization from vaccinated and naïve control mice (*n =* 5 per group). The isolated splenocytes were stimulated with rF1V (100 µg/mL) for 48 h at 37 °C, followed by an additional 4 h with Brefeldin A. T cells were then harvested and stained with specific T cell AIM markers for flow cytometry analysis. The percentages of positive AIM populations in (**A**) CD4^+^CD44^+^CD134^+^CD25^+^ and (**B**) CD8^+^CD44^+^CD69^+^CD25^+^ were acquired. The arithmetic means ± standard deviations are plotted. Statistical significance was determined using one-way ANOVA with Tukey’s post hoc test. Asterisks with comparison bars represent statistical significance between respective vaccinated groups, while asterisks directly above the bars represent the statistical significance of vaccinated groups compared to naïve control mice. * *p* < 0.05, ** *p* < 0.01, *** *p* < 0.001, ****, *p* < 0.0001.

We also utilized an activation-induced marker (AIM) assay to further examine antigen-activated T cells that might not express sufficient cytokines to be measured by traditional intracellular staining (ICS) methods [45]. These T cells were antigen-experienced (CD44^+^) and were further gated with CD134^+^CD25^+^ for CD4 or CD69^+^CD25^+^ for CD8. Overall, vaccinated mice displayed increased antigen-activated T cells compared to unvaccinated control animals although some did not reach statistically significant levels (Figure 8A,B). Levels of antigen-activated T cells were generally equivalent between immunized IL-17A-depleted and IgG control mice, except for CD8^+^ cells from LMP–Ad5-YFV-immunized mice, which were significantly lower in IL-17A-depleted mice.

**Figure 9:**
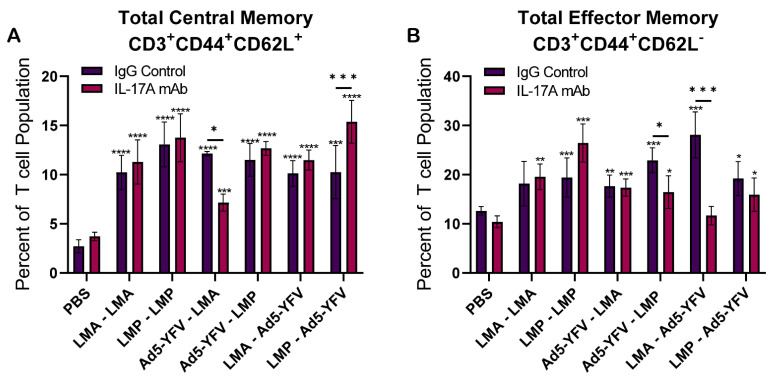
Memory T cell populations in vaccinated IL-17A-depleted and IgG control mice. Spleens were collected 4 weeks post-boost immunization from vaccinated and naïve control mice (*n =* 5 per group). The isolated splenocytes were stimulated with rF1V (100 µg/mL) for 48 h at 37 °C, followed by an additional 4 h with Brefeldin A. Cells were then harvested and stained with various T cell markers and subjected to flow cytometry. (**A**) Central memory T cells (TCM) were identified as CD44^+^CD127^+^CD62L^+^, while (**B**) effector memory T cells (TEM) were identified as CD44^+^CD127^+^CD62L^-^. The arithmetic means ± standard deviations are plotted. Statistical significance was determined using one-way ANOVA with Tukey’s post hoc test. Asterisks with comparison bars represent statistical significance between respective vaccinated groups, while asterisks directly above the bars represent the statistical significance of vaccinated groups compared to naïve control mice. * *p* < 0.05; ** *p* < 0.01; *** *p* < 0.001; **** *p* < 0.0001.

We next evaluated central (TCM) and effector (TEM) memory T cell responses. TCM cells interact with antigen-specific dendritic cells in primary lymphoid tissues before expansion to acquire effector functions. TEM cells migrate into secondary lymphoid tissues to interact with pathogens directly and provide cytotoxic functions [46]. Compared to unimmunized control animals, the populations of both TCM and TEM cells generally increased after vaccination, although a few did not reach significant levels (Figure 9A,B). However, comparable levels of memory T cells were observed across the majority of IL-17A-depleted and IgG control groups in response to vaccination. Significant differences were only observed in the Ad5-YFV–LMA- or LMP–Ad5-YFV-immunized groups for TCM and in Ad5-YFV–LMP- or LMA–Ad5-YFV-immunized groups for TEM, respectively (Figure 9A,B).

## 4. Discussion

IL-17 family cytokines exhibit diverse biological functions that promote multi-pathogen protective immunity and drive autoimmune and infection-related inflammation [47]. The IL-17 family consists of six members, from IL-17A to F, based on sequence homology, with IL-17A and IL-17F sharing the greatest homology (50–55%) between them. The corresponding genes of IL-17A and IL-17F are located in close proximity on the same chromosome in both humans and mice [39,48,49]. IL-17 signaling is initiated by cell membrane receptors composed of five members (IL-17RA to IL-17RE), which function either as homodimeric or heterodimeric proteins characterized by the conserved intracellular domain SEFIR (similar expression to fibroblast growth factor genes) [50]. IL-17A, the most widely studied and significant member of the IL-17 family, forms either homodimers alone or heterodimers with IL-17F and binds to a receptor complex of IL-17RA/IL-17RC. These mediate biological and immunological functions mainly through activation of the nuclear factor-kappa B (NF-kB) signaling pathway [48,51]. 

Th17 cells are the main source of IL-17 family cytokines; however, these cytokines can also be produced by innate immune cells in response to IL-1β and IL-23 stimulation [47]. The IL-17 pathway is currently a strategic drug target for many autoimmune and chronic inflammatory disorders. Therapeutic mAbs target IL-17A and F, their receptors, or IL-23, an important cytokine for maintaining the differentiation state of Th17 cells that produce IL-17A and IL-17F. Indeed, antibodies against the above targets are exceedingly effective in many diseases [47,51]. On the other hand, fungal and bacterial protective immunity is mediated by IL-17, further promoting neutrophil recruitment, antimicrobial peptide production, and maintaining epithelial barrier function [47,48,51]. Importantly, evidence also suggests that Th17 is critical for vaccine-induced immune responses against infectious diseases, especially at the mucosal surface [52,53].

For example, IL-23 and IL-17A have been shown to play a critical role in the expression of vaccine-induced immunity against pulmonary tuberculosis [54]. Furthermore, Bacille Calmette Guerin (BCG) vaccination followed by boosting with either viral vector- or plasmid-based mycobacterial protective antigen 85 (Ag85A) elicited greater expression of IL-17A, correlating with better protection compared to BCG vaccination alone in mice and cattle [55,56]. Interestingly, IL-17A was not detectable in mice primed with DNA Ag85A or vaccinated with BCG alone [55]. The lack of protection against pulmonary tuberculosis further suggests that the generation of IL-17A through vaccination may be a prerequisite for the development of a successful long-lasting memory immune response [52]. Likewise, the role of IL-17A in vaccine-induced responses against extracellular bacteria such as *Streptococcus pneumoniae*, *Bordetella pertussis*, *Helicobacter pylori*, and *Pseudomonas aeruginosa* has also been established [52].

Th17 cells are effective helpers for B cells, and blockade of IL-17A signaling significantly reduces germinal centers (GCs) in both number and size, driving B cell isotype class switching to IgG2a and IgG3 subtypes [57]. In our study, overall enhanced IgG titers to the F1V fusion protein with a generally balanced Th1/Th2 response were observed in all immunized mice, and animals receiving LAVs as the first dose had slightly higher IgG1 over IgG2a compared to other immunization strategies (Figure 2). A similar phenomenon was also observed in our previous study [40]. In addition, both immunized IL-17A-depleted and IgG control mice had comparable levels of GCs and memory B cells (Figure 6). However, significantly higher serum IgG titers to either F1V or F1 were noted in immunized IL-17A-depleted mice. In contrast, antibody titers to YscF were significantly lower in immunized mice when IL-17A was depleted (Figure 2 and Figure 3). Clearly, the class switching of IgG isotypes, GC activities, and serum IgG titers appeared to be more associated with antigen-specific response, or type of vaccine, rather than IL-17A depletion. The differences between this study and a previous study [57] could be related to different stimulating antigens, mouse strains, and immunological methods used. In our study, we utilized Swiss Webster outbred mice, LAVs alone or in combination with Ad5-YFV, and flow cytometry for GC activities, while others utilized *in vitro* assays along with C57BL/6 mice, myelin oligodendrocyte glycoprotein (MOG35-55) peptide, and immunofluorescence staining for GCs [57]. 

The specific reasons for the opposite antibody titer patterns to F1 and YscF in IL-17A-depleted mice are not clear; however, intertwined roles and functions of IL-17A and IL-17F are plausible. In certain clinical cases, targeting IL-17A alone may not be enough to control disease long term [58,59,60,61,62]; therefore, inhibition of both IL-17A and F might be preferable [39]. Activated Th17 cells also differ in the subtype of IL-17 that they preferentially express: IL-17A, IL-17A/F, or IL-17F. Th17 differentiation is typically controlled by three factors: cytokine environment, strength or concentration of antigenic signaling through the T cell receptor (TCR), and stimulus duration [63,64]. For example, the IL-17A^+^ subpopulation is preferentially promoted through low-strength T cell activation, whereas the IL-17F^+^ subpopulation is preferentially promoted through high-strength stimulation [39]. Therefore, differing antibody titers to F1 and YscF could be related to the strengths associated with either IL-17A^+^ or IL-17F^+^ subpopulations. 

Interestingly, compared to immunized IgG control mice, overall IgA titers to all three examined antigens were either at comparable levels or lower in IL-17A-depleted mice (Figure 3), suggesting the influence of IL-17A was more obvious in production of mucosal IgA rather than serum IgG. This could be related to a cytokine environment that is more favorable to IL-17A^+^ subpopulations in the mucosa, providing evidence that Th17 is more critical for vaccine-induced immune responses against mucosal infectious diseases [52,54]. It must be mentioned that induction of an IL-17A response was only observed in mice immunized with either our LAVs alone or in combination with Ad5-YFV and was not seen in mice vaccinated with Ad5-YFV alone [9,40]. Therefore, unidentified antigens in our LAVs could potentially be responsible for the induction of IL-17A, and F1, LcrV, and YscF may not play a significant role. Nevertheless, our vaccines induced strong humoral and mucosal immunity in mice irrespective of IL-17A depletion. 

Neutralizing antibodies are integral in directly inhibiting the transmission or infectivity of pathogens by interacting with specific epitopes on antigens [65]. Studies have shown that certain epitope-specific antibodies restrict the mobility of *P. aeruginosa* [66], *Vibrio cholerae* [67], and *Salmonella enterica* [68]**,** thereby decreasing their ability to infect host cells. mAbs targeting the *Y. pestis* F1 capsular antigen have been identified, exhibiting high binding affinity, epitope specificity, and protective efficacy against bubonic and pneumonic challenges [69,70]. In addition, two mAbs targeting *Y. pestis* LcrV, 7.3 and 29.3, were shown to have high binding affinity and neutralizing activity [44]. Mab7.3 binds to the region spanning amino acids 135–275 and, while much less is known about Mab29.3, it binds to a region near the Mab7.3 epitope on LcrV [44]. While 50 μg LcrV Mab7.3 administered 24 h post-bubonic challenge provided 100% protection to mice, administration of 50 μg LcrV Mab29.3 provided 80% protection [44]. Furthermore, the protective efficacy of Mab7.3 has also been demonstrated in different species of mice infected with various *Y. pestis* strains, including CO92, in both bubonic and pneumonic plague models [44]. Humanized Mab7.3 is currently being produced for therapeutic usage [44].

To quantify neutralizing antibodies in the sera of immunized mice, competitive ELISAs with biotinylated LcrV Mab7.3 were utilized. Higher levels of LcrV-neutralizing antibodies were detected in all immunized mice compared to unimmunized controls (Figure 4). Heterologous vaccination, where Ad5-YFV was administered first, as opposed to the LAVs, elicited slightly higher LcrV-neutralizing antibodies (Figure 4). This could be due to conformational differences in LcrV between the LAVs and the adenovirus-based YFV fusion protein. The LcrV epitope recognized by Mab7.3 could be better presented by antigen-presenting cells (APCs) in Ad5-YFV rather than in its natural form within the LAVs. In addition, access to LcrV on the surface of *Y. pestis* could also be masked by other bacterial components, such as the F1 capsule. Therefore, the clear presentation of LcrV epitopes by Ad5-YFV would eventually lead to a better antibody response. As also noted in Figure 4, neutralizing antibody titers were higher in vaccinated IgG controls compared to IL-17A-depleted animals, in contrast to the comparable serum LcrV antibody titers between these groups (Figure 3). The strength of antigen epitopes may also determine their association with either IL-17A^+^ or IL-17F^+^ subpopulations, as discussed for F1 and YscF above. 

Compared to vaccine-induced antibody production, a clearer trend was observed for the influence of IL-17A on T cell responses during immunization. More specifically, T cells positive for IL-17A, IFNγ, and IL-2 were all significantly lower in IL-17A-depleted mice (Figure 7A–F). Impaired IFNγ production was also evidenced in mycobacterial-specific T cells from IL-17A-deficient mice [71]. The frequency of Ag-specific IFNγ–producing lung T cells and Th1 cell recruitment was significantly reduced when IL-17A was neutralized in vaccinated mice during *Mycobacterium tuberculosis* challenge [54]. 

Similarly, IL-17A is required for the generation of protective IFNγ responses during pulmonary tularemia [72]. The correlation of IL-17A- and IFNγ-producing T cells seen in various bacterial infections may be related to the *in vivo* plasticity of Th17 cells, where cytokine production can switch from predominantly producing IL-17 to IFNγ, thereby resembling Th1 cells [47,73]. In addition, IL-4 suppresses the reactivation of committed Th17 cells, even in the presence of TGF-β, IL-6, and IL-23. Further, signal transducer and activator of transcription 6 (STAT6)-dependent IL-4 down-regulation of IL-17A is mediated by inhibition of STAT3 binding at the IL-17A promoter [74]. Although IFNγ expression by Th17 cells is induced by Th1 cytokines, IL-4 does not induce a Th2 phenotype in Th17 cells. As such, a correlation between IL-17A and IFNγ was not observed with the Th2 cytokine IL-4 [74]. Indeed, our data suggested that the influence of IL-17A on IL-4^+^ T cell populations seemed to depend on the type of vaccine the animals received and there was no clear pattern related to IL-17A depletion (Figure 7I–J).

The significant reduction in Th1^+^ T cells in IL-17A-depleted mice prompted us to further evaluate overall T cell activation using the AIM assay, based on T cell receptor-stimulated surface markers [45]. These markers determine overall T cell activation, rather than only specific cytokine markers utilized with traditional staining techniques [75]. While there were no differences between IL-17A-depleted and IgG control animals, vaccinated mice displayed an overall increase in activated antigen-specific T cells (Figure 8). A similar pattern was observed for memory T cells as their overall levels were comparable in both immunized IL-17A-depleted and IgG control animals (Figure 9A,B). 

## 5. Conclusions

In summary, the depletion of IL-17A clearly demonstrated impacts on both humoral and cellular immune responses elicited by our vaccines; however, the overall immunity associated with vaccination in IL-17A-depleted mice was adequate to protect them from lethal *Y. pestis* CO92 challenge. Our earlier study also demonstrated that heterologous vaccination with Ad5-YFV and LMA protected mice against lethal challenge with the non-encapsulated *Y. pestis* (F1^-^) CO92 strain [40]; therefore, a similar study in IL-17-depleted mice will be conducted in the future to further evaluate the efficiency of our vaccines against different strains of *Y. pestis*. In addition to promoting neutrophil recruitment, producing antimicrobial peptides, and maintaining epithelial barrier function, IL-17 is also closely associated with host defense responses by promoting tertiary immunity in lymphoid tissues [38]. For example, it promotes bronchus-associated lymphoid tissue immunity against the intracellular pathogen *M. tuberculosis* [54,76]. Furthermore, studies in a *B. pertussis* infection mouse model showed that antigen-specific Th17 cells in nasal tissue predominantly produced IL-17 without IFNγ and persisted as tissue-resident memory T (TRM) cells for many months after bacterial clearance [77]. Therefore, the evaluation of local lung immunity, e.g., local B and T cell levels in response to our vaccines in both IL-17A and IL-17F depleted animals, could shed more light on the role of IL-17 in modulating acquired immunity. 

It is worth pointing out that several recent studies have reported the effects of sex-based differences on the efficacy of plague vaccines, where female mice generated better immunity in response to vaccination than males [78,79]. Therefore, our future studies will use animals balanced by sex, as in our recently published phage therapy study for *Y. pestis* infection [80]. We must emphasize that the conclusions of this study originate from a mouse model. While the biological functions of murine and human IL-17 are very similar [47,81], subtle differences in IL-17-related pathways, including IL-17 ligand–receptor interactions [82], IL-17-producing cell type differentiation and stabilization [83], and genetic diversity [84,85], have been observed between mice and humans. Therefore, evaluation of our vaccines in higher animal models, as we previously conducted for Ad5-YFV in nonhuman primates [10], is warranted and will be included in our future studies. 

## Data Availability

The original contributions presented in this study are included in the article. Further inquiries can be directed to the corresponding authors.
